# Letter to the Editor: Homeopathic drug-induced liver injury—an example of biases pertaining to Roussel Uclaf causality assessment method

**DOI:** 10.1097/HC9.0000000000000177

**Published:** 2023-06-14

**Authors:** Arun Krishnan P., Muraleedharan K. Charan

**Affiliations:** Department of Central Council for Research in Homoeopathy, National Homoeopathy Research Institute in Mental Health, Kottayam, Kerala

To the editor

The study published by Theruvath et al^1^ showed some serious flaws in data capturing and depiction. The authors should have depicted the data appropriately, especially concerning the Roussel Uclaf Causality Assessment Method (RUCAM) and observations related to gas chromatography-mass spectroscopy analysis (GC/MS).

## OBSERVATIONS RELATED TO RUCAM

The causality assessment was done using the updated RUCAM.^2^ The following violations of the method were observed in patient data:

1. Liver injury is defined as serum levels of alanine transferase (ALT) of at least 5N (N is the upper limit of the reference range) and/or serum alkaline phosphatase (ALP) levels of at least 2N.^2^ This threshold has been adopted by updated RUCAM to negate false-positive cases. When the standard reference ranges for ALT (10–49 IU/L) and ALP (46–116 IU/L) values are considered for inclusion, ALT should be ≥245 IU/L and ALP should be ≥232 IU/L. Based on this reference, 4 patients do not have even liver injury (Supplemental Table S1, http://links.lww.com/HC9/A315).
2. For estimating the dechallenge course, the values of ALT on the eighth and 30th days and ALP on the 180th day are required,^2^ which are not provided. If it is not done, a score of zero may be assigned to this domain.
3. Any intervention during the dechallenge phase may mask the natural course of dechallenge. Therefore, at least 5 patients can be given only a score of “0” for this domain, owing to the administration of corticosteroid and ursodeoxycholic acid (Supplemental Table S2, http://links.lww.com/HC9/A316).
4. If any patients receive any comedication or dietary supplements, individual RUCAM assessment needs to be done for those comedications, which was not done in 4 patients. It should also be noted that the Methods section^1^ has explained that those patients taking comedications were excluded. Still, the analysis includes them (Supplemental Table S3, http://links.lww.com/HC9/A317).
5. Operational information on updated RUCAM has mentioned that it applies only to those with acute liver injury and is not intended for patients with pre-existing chronic liver diseases.^2^ However, underlying hepatic disorders were reported in 6 patients (Supplemental Table S4, http://links.lww.com/HC9/A318). A score of “-3” may be assigned if such alternative causes are highly probable.
6. For estimating the rechallenge course, details about the baseline or re-exposure values of ALT are required. However, these values are unavailable, despite an intentional re-exposure.
7. The histopathology data are irrelevant for diagnosing DILI as per the updated RUCAM,^2^ as it has many similar features of primary hepatic or biliary diseases.^3^ However, in the discussion part,^1^ authors are trying to substantiate that the liver histopathologies identified are supposed to be due to homeopathic drugs.

The authors recalculated the RUCAM score based on the aforementioned observations and found that 4 patients out of 9 must be excluded. Of the 5 patients included, 4 are under the “unlikely” and 1 under the “possible” category (Supplemental Table S5, http://links.lww.com/HC9/A319). RUCAM warrants transparent reporting of baseline and final scores for each patient and product for appropriate causality assessment. Unfortunately, the discrepancies cited clearly indicate the inadequacy of the data, making the study findings invalid.

## OBSERVATIONS RELATED TO GC/MS ANALYSIS

1. The data on GC/MS analysis failed to provide any quantitative information about any of the components in terms of units of measurement.
2. The only components identified in some medicines were simple fatty acids and carbohydrates (Supplemental Table S6, http://links.lww.com/HC9/A320), the edibility of which is well known.
3. Any substance can be a potential poison, and dose is one of the critical factors deciding the toxicity of a substance. Unfortunately, no supporting documents have been provided to define the exposure (homeopathic remedies) detailing their prescription, doses, and frequency, which would have supported the claim of DILI.

Altogether the article has shown enough examples of selection and information biases with a weak causality link between homeopathic remedies and DILIs. From the overall review, it is understood that the conclusions derived from the given article^1^ are irrelevant.

## Supplementary Material

## References

[1] Theruvath AH Raveendran R Philips CA Ahamed R Abduljaleel JK Tharakan A. A series of homeopathic remedies-related severe drug-induced liver injury from South India. Hepatol Commun. 2023;7:e0064. 36757412 10.1097/HC9.0000000000000064 PMC9916127

[2] Danan G Teschke R. RUCAM in drug and herb induced liver injury: the update. Int J Mol Sci. 2015;17:14. 26712744 10.3390/ijms17010014 PMC4730261

[3] Teschke R Frenzel C. Drug induced liver injury: do we still need a routine liver biopsy for diagnosis today? Ann Hepatol. 2014;13:121–6. 24378275

