# Supplementary material for: Letter to the Editor: Homeopathic drug-induced liver injury—an example of biases pertaining to Roussel Uclaf causality assessment method
Source: Hepatol Commun. 2023 Jun 14;7(7):e00177. doi: 10.1097/HC9.0000000000000177 (PMC10270482; doi:10.1097/HC9.0000000000000177)
Supplement: Supplementary file 1 [file hc9-7-e00177-s001.docx]

**Manuscript ID HEP4-23-0262**

**Supplementary Table 1:** The list of patients having low Alanine Amino Transferase(ALT) and Alkaline Phosphatase(ALP) values^1^

| **Sl. No. of the patient** | **Age/Sex** | **ALT(IU/L)** | **ALP(IU/L)** |
| --- | --- | --- | --- |
| 1 | 65/F | 82 | 152 |
| 4 | 54/M | 54 | 127 |
| 7 | 70/M | 161 | 212 |
| 9 | 38/M | 82 | 118 |

Footnote: Sl. No.- Serial Number, F- Female, M-Male, ALT- Alanine Amino Transferase(IU/L), ALP-Alkaline Phosphatase(IU/L).
