# Supplementary material for: Letter to the Editor: Homeopathic drug-induced liver injury—an example of biases pertaining to Roussel Uclaf causality assessment method
Source: Hepatol Commun. 2023 Jun 14;7(7):e00177. doi: 10.1097/HC9.0000000000000177 (PMC10270482; doi:10.1097/HC9.0000000000000177)
Supplement: Supplementary file 2 [file hc9-7-e00177-s002.docx]

**Manuscript ID HEP4-23-0262**

**Supplementary Table 2:** The list of patients who have taken treatment during dechallenge period^1^

| **Sl. No. of the patient** | **Age/Sex** | **Name of the drugs** |
| --- | --- | --- |
| 1. | 65/F | 1. Albumin infusion, 2.Empirical antibiotics, 3.Intravenous N-acetyl cysteine  4.Intravenous glutathione |
| 2. | 26/F | 1. Weight-based corticosteroids for one month 2.Ursodeoxycholic acid 10mg/kg for one month |
| 3. | 54/F | 1. Weight-based corticosteroids tapered and stopped over six months  2. N-acetyl cysteine 1.2g/d for one month |
| 4. | 54/M | 1. Albumin infusion  2. Empirical antibiotics  3. Intravenous N-acetyl cysteine  4. Ursodeoxycholic acid (10 mg/kg per day in divided dose) |
| 5. | 27/M | 1. N-acetyl cysteine  2. Oral glutathione for one month |
| 6. | 68/M | 1. Immunosuppression – corticosteroid (first 6 months)  2. Azathioprine after 6 months, continued, low dose |
| 7. | 70/M | 1. Albumin infusion  2. Empirical antibiotics  3. Intravenous N-acetyl cysteine  4. Ursodeoxycholic acid (10 mg/kg per day in divided dose) |
| 8. | 34/M | 1. Corticosteroids (weight based)  2. Intravenous and oral N-acetyl cysteine  3. Ursodeoxycholic acid (10 mg/kg per day in divided dose)  4. Intravenous and oral glutathione1 |
| 9. | 38/M | 1. A short course of corticosteroids (stopped after two weeks)  2. Albumin infusion  3. Empirical antibiotics  4. Intravenous N-acetyl cysteine  5. Ursodeoxycholic acid (10 mg/kg per day in divided dose) |

Footnote: Sl. No.- Serial Number, F- Female, M-Male
