# Supplementary material for: Letter to the Editor: Homeopathic drug-induced liver injury—an example of biases pertaining to Roussel Uclaf causality assessment method
Source: Hepatol Commun. 2023 Jun 14;7(7):e00177. doi: 10.1097/HC9.0000000000000177 (PMC10270482; doi:10.1097/HC9.0000000000000177)
Supplement: Supplementary file 3 [file hc9-7-e00177-s003.docx]

**Manuscript ID HEP4-23-0262**

**Supplementary Table 3:** The list of patients taking concomitant drugs^1^

| **Sl. No. of the patient** | **Age/Sex** | **Concomitant drugs taken** |
| --- | --- | --- |
| 4 | 54/M | 1. Beta blockers  2. Low dose diuretics  3. Vitamin E supplements  4. Vitamin supplements  5. Lactulose syrup  All these drugs were ongoing for more than a year. |
| 6 | 68/M | 1. Vitamin E supplements  2. Telmisartan |
| 7 | 70/M | 1. Metformin  2. Glimepride  3. Acarbose  The patient was under these medications for 5 years. |
| 9 | 38/M | 1. Vitamin E supplements  2. Multivitamin B complex tablets  Patient was on these medications 4 months before the start of Homoeopathy. |

Footnote: Sl. No.- Serial Number, F- Female, M-Male
