# Supplementary material for: Letter to the Editor: Homeopathic drug-induced liver injury—an example of biases pertaining to Roussel Uclaf causality assessment method
Source: Hepatol Commun. 2023 Jun 14;7(7):e00177. doi: 10.1097/HC9.0000000000000177 (PMC10270482; doi:10.1097/HC9.0000000000000177)
Supplement: Supplementary file 4 [file hc9-7-e00177-s004.docx]

**Manuscript ID HEP4-23-0262**

**Supplementary Table 4:** The list of patients having underlying liver diseases^1^

| **Sl. No. of the patient** | **Age/ Sex** | **Underlying liver diseases** |
| --- | --- | --- |
| 1. | 65/F | 1. Non Alcoholic Fatty Liver Disease  2. Liver Carcinoma |
| 4. | 54/M | 1. Acute on Chronic Liver Failure |
| 5. | 27/M | 1. Gall bladder stones |
| 6. | 68/M | 1. Non Alcoholic Fatty Liver Disease |
| 7. | 70/M | 1. Non Alcoholic Fatty Liver Disease |
| 9. | 38/M | 1. Non Alcoholic Fatty Liver Disease  2. Gilbert’s syndrome  3. Cirrhosis |

Footnote: Sl. No.- Serial Number, F- Female, M-Male
