# Supplementary material for: Letter to the Editor: Homeopathic drug-induced liver injury—an example of biases pertaining to Roussel Uclaf causality assessment method
Source: Hepatol Commun. 2023 Jun 14;7(7):e00177. doi: 10.1097/HC9.0000000000000177 (PMC10270482; doi:10.1097/HC9.0000000000000177)
Supplement: Supplementary file 5 [file hc9-7-e00177-s005.docx]

**Manuscript ID HEP4-23-0262**

**Supplementary Table 5:** New total RUCAM score for the patients, obtained based on observations

| **Sl.**  **No. of the patient** | **Age**  **/Sex** | **Included/**  **Excluded** | **Reason for exclusion** | **New RUCAM score** |
| --- | --- | --- | --- | --- |
| 1. | 65/F | Excluded | 1. No liver injury as ALT<5N and ALP <2N | NA |
| 2. | 26/F | Included | NA | 1. Time to onset:+2  2. Course of reaction:0  3. Risk factors:0  4. Concomitant drugs:0  5. Search for alternative causes:0  6. Previous hepatotoxicity of the drug:0  7.Response to unintentional exposure: +3(Even though no data)  Total score: 5(possible) |
| 3. | 54/F | Included | NA | 1. Time to onset:+2  2. Course of reaction:0  3. Risk factors:0  4. Concomitant drugs:0  5. Search for alternative causes:0  6. Previous hepatotoxicity of the drug:0  7. Response to unintentional exposure:0  Total score:2(unlikely) |
| 4. | 54/M | Excluded | 1. No liver injury as ALT<5N and ALP <2N | NA |
| 5. | 27/M | Included | NA | 1. Time to onset:+2  2. Course of reaction:0  3. Risk factors:0  4. Concomitant drugs:0  5. Search for alternative causes:0  6. Previous hepatotoxicity of the drug:0  7. Response to unintentional exposure:0  Total score:2(unlikely) |
| 6. | 68/M | Included | NA | 1. Time to onset:+2  2. Course of reaction:0  3. Risk factors:+1  4. Concomitant drugs:-1  5. Search for alternative causes:-3  6. Previous hepatotoxicity of the drug:0  7. Response to unintentional exposure:0  Total score:-1(unlikely) |
| 7. | 70/M | Excluded | 1. No liver injury as ALT<5N and ALP <2N | NA |
| 8. | 34/M | Included | NA | 1. Time to onset:+2  2. Course of reaction:0  3. Risk factors:0  4. Concomitant drugs:0  5. Search for alternative causes:0  6. Previous hepatotoxicity of the drug:0  7. Response to unintentional exposure:0  Total score:2(unlikely) |
| 9. | 38/M | Excluded | 1. 1.No liver injury as ALT<5N and ALP <2N | NA |

Footnote: Sl. No.- Serial Number, F- Female, M-Male, ALT- Alanine Amino Transferase(IU/L), N-Upper Limit of Normal Range, ALP-Alkaline Phosphatase(IU/L), NA- Not Applicable
