# Supplementary material for: Letter to the Editor: Homeopathic drug-induced liver injury—an example of biases pertaining to Roussel Uclaf causality assessment method
Source: Hepatol Commun. 2023 Jun 14;7(7):e00177. doi: 10.1097/HC9.0000000000000177 (PMC10270482; doi:10.1097/HC9.0000000000000177)
Supplement: Supplementary file 6 [file hc9-7-e00177-s006.docx]

**Manuscript ID HEP4-23-0262**

**Supplementary Table 6:** List of patients and their implicated drugs with three or less than three compounds identified^1^

| **Sl. No. of the given patient** | **Age/Sex** | **Code/Name of the drug** | **Concomitant drugs** | **Compounds identified** | **RUCAM score** |
| --- | --- | --- | --- | --- | --- |
| 3 | 54/F | D2(Arsenic Album 30 in globules) | None | 1. Palmitin, 1,2-di(Fatty acid) | 8 |
| 4 | 54/M | D1(Unlabelled globules) | 1. Beta blockers 2.Low-dose diuretics 3.Vitamin E supplements  4.Vitamin B supplements  5.Lactulose syrup  These drugs were being taken since 1 year. | 1.d mannose(Monosacharide)  2.melezitose(Trisacharides) | 6 |
| 6 | 68/M | D2(Arsenic Album 30) | 1. Vitamin E supplements 2.Telmisartan | 1.palmitin,1,2-di(Fatty acid) | 7 |

Footnote: Sl. No.- Serial Number, F- Female, M-Male
